# Decadal climate predictability in the southern Indian Ocean captured by SINTEX-F using a simple SST-nudging scheme

**DOI:** 10.1038/s41598-018-19349-3

**Published:** 2018-01-26

**Authors:** Yushi Morioka, Takeshi Doi, Swadhin K. Behera

**Affiliations:** Application Laboratory, JAMSTEC, Yokohama, Japan

## Abstract

Decadal climate variability in the southern Indian Ocean has great influences on southern African climate through modulation of atmospheric circulation. Although many efforts have been made to understanding physical mechanisms, predictability of the decadal climate variability, in particular, the internally generated variability independent from external atmospheric forcing, remains poorly understood. This study investigates predictability of the decadal climate variability in the southern Indian Ocean using a coupled general circulation model, called SINTEX-F. The ensemble members of the decadal reforecast experiments were initialized with a simple sea surface temperature (SST) nudging scheme. The observed positive and negative peaks during late 1990s and late 2000s are well reproduced in the reforecast experiments initiated from 1994 and 1999, respectively. The experiments initiated from 1994 successfully capture warm SST and high sea level pressure anomalies propagating from the South Atlantic to the southern Indian Ocean. Also, the other experiments initiated from 1999 skillfully predict phase change from a positive to negative peak. These results suggest that the SST-nudging initialization has the essence to capture the predictability of the internally generated decadal climate variability in the southern Indian Ocean.

## Introduction

Decadal climate variability in the southern Indian Ocean plays an important role in decadal climate variation over southern Africa through changes in atmospheric circulation^1^. Observation during recent decades shows that warm sea surface temperature (SST) anomalies in the southwest Indian Ocean are accompanied by high sea level pressure (SLP) above, and the associated easterly wind anomalies bring more moisture toward southern Africa, leading to rainfall increase^2,3^. It was widely accepted that local air-sea interaction in the southern Indian Ocean^4,5^ and remote forcing due to modulation of the Indonesian Throughflow^6^, probably through changes in the equatorial-subtropical current system^7^, are responsible for generating the decadal climate variability. Some of the recent studies^8–10^ have suggested another remote influence arising from the SST signal in the South Atlantic that propagates to the southern Indian Ocean. Since the decadal climate variability has long-term socio-economic impacts over southern Africa through consecutive droughts or floods, skillful prediction of decadal climate variability in the southern Indian Ocean is indispensable.

Prediction of decadal climate variability has extensively been performed under the international research framework of World Climate Research Program (WCRP), called the Decadal Climate Prediction Project (DCPP). Major finding and challenges of decadal climate prediction are briefly described in a review paper^11^. Most studies have focused on identifying predictability of decadal variation in the global surface temperature^12,13^ and/or in specific ocean basins such as the tropical Pacific^11,14,15^, the North Pacific^16,17^ and the North Atlantic^18–20^. However, a few studies have detailed predictability of decadal climate variation in the southern Indian Ocean. For example, Guemas *et al*.^21^ discussed that prediction skill of the surface temperature in the Indian Ocean is the highest among the global oceans due to the externally forced trend from increasing greenhouse gasses, but prediction skills in the southern Indian Ocean are relatively low due to the internally generated variability.

Predictability of the internally generated climate variability has extensively been studied for interannual oceanic climate phenomena such as El Niño/Southern Oscillation (ENSO) and the Indian Ocean Dipole (IOD)^22^. Among many studies, Luo *et al*.^23,24^ using a state-of-the-art coupled general circulation model (CGCM) performed hindcast experiments with a simple SST-nudging initialization scheme in which the model SST is strongly relaxed to the observation using surface heat flux, and showed skillful predictions for ENSO up to 1-yr ahead and the IOD to 3–4 months ahead. Although prediction skills are relatively low in the extratropics, follow-up studies^25,26^ using the same SST-nudging scheme did demonstrate skillful predictions of interannual ocean-climate phenomena in the South Atlantic and southern Indian Ocean; called Subtropical Dipoles^27,28^. Considering existence of internally generated climate variability in the extratropics, the SST-nudging scheme may be helpful not only for seasonal climate prediction^29–31^ but also for the decadal climate prediction^18^.

In this context, the present study aims at identifying predictability of decadal climate variation in the southern Indian Ocean using a state-of-the-art CGCM with a simple SST-nudging initialization scheme. The paper is organized as follows: comparison between the observation and decadal reforecast results are made in Results section, and limitation of decadal climate prediction using the SST-nudging scheme and further improvement of model experiments and initialization scheme are described in Discussion section. A brief description of observational data, CGCM, and decadal reforecast experiments is given in Methods section.

## Results

### Predictability in the southern Indian Ocean

**Figure 1:**
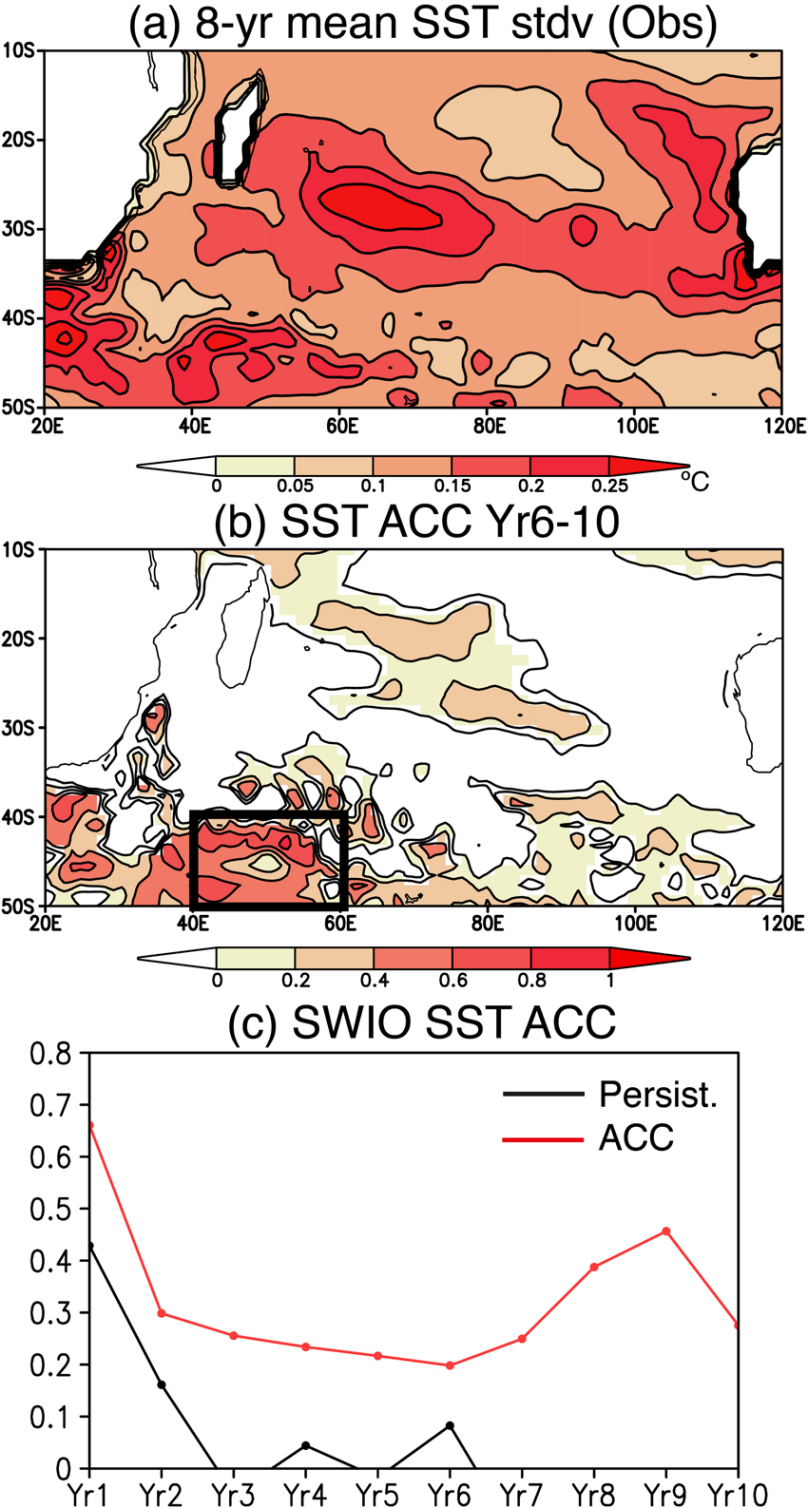
(**a**) Standard deviation of decadal SST anomalies (°C) in the observation. 8-yr running mean was applied to calculate decadal SST anomalies. (**b**) Anomaly correlation coefficient (ACC) of the 5-yr mean SST anomalies between the observation and reforecast experiments over 6–10 year lead times. The significant ACCs above the persistence values are colored. Here we used 12 ensemble mean SST anomalies for the model results. (**c**) Persistence (black line) and the ACC (red line) of the yearly mean SWIO index for each year lead time. The SWIO index is defined as the SST anomalies averaged in the southwest Indian Ocean (40–60°E and 40–50°S, black box in (**b**)). The maps were generated using Grid Analysis and Display System (GrADS) Version 2.1.a3 (http://cola.gmu.edu/grads/downloads.php).

Decadal variability in the southern Indian Ocean is well pronounced east of Madagascar, off the west coast of Australia, and in the Agulhas Retroflection region. This can be seen in the observed standard deviation of 8-yr low-pass filtered SST anomalies (Fig. 1a). Prediction skills of decadal climate variation in the southern Indian Ocean were further estimated using anomaly correlation coefficient (ACC) between yearly mean SST anomalies derived from the observation and the ensemble mean of reforecast results. Here we used yearly mean anomalies without detrending in order to calculate the ACC. Figure 1b shows a spatial pattern of the ACC between observed and model SST anomalies predicted over 6–10 year lead times. The ACC is significantly high in the southwest Indian Ocean (40–60°E and 40–50°S), where the decadal variability is pronounced (Fig. 1a). The zonally elongated structure seen in the latitudinal band of 40–50°S indicates high prediction skills of SST variability along strong eastward currents such as the Agulhas Return Current and the Antarctic Circumpolar Current. The ACC east of Madagascar is also significant, but the amplitude is much weaker than the ACC in the southwest Indian Ocean.

The high ACC in the southwest Indian Ocean is further examined by defining SWIO index as the SST anomalies averaged over the southwest Indian Ocean (black box in Fig. 1b). Figure 1c shows the ACC of the SWIO indices between the observation and the ensemble mean reforecast results at each year lead time. From prediction year 1 to year 10, the ACCs show significant values, exceeding persistence values defined as autocorrelation coefficient of the observed SST anomalies. The ACC of the SWIO index sharply drops from year 1 to year 2, and has insignificant values of 0.2–0.3 through to year 6, thereafter it revives to about 0.5 around year 9. This corresponds to the high values of the ACCs seen in the southwest Indian Ocean for 6–10 year lead times (Fig. 1b).

The revival of the ACC of the SWIO index at prediction year 9 could be associated with a predictable decadal SST variability in that region. Previous studies^2,3,8,10^ show a clear bi-decadal variability in the southwest Indian Ocean with frequency of 18–20 years, so every 9–10 years the SST in the southwest Indian Ocean experiences a wave train of positive and negative anomalies propagating from the South Atlantic to the southern Indian Ocean. Since the decadal reforecast experiments adopted SST-nudging scheme for initialization, the predictable decadal SST signal in the southwest Indian Ocean was to some extent embedded in that eastward propagation of the initialized SST anomalies from the South Atlantic. Although the ACC of the South Atlantic SST in the same latitudinal band (40–50°S) is lower than that of the SST in the southwest Indian Ocean (see Supplementary Fig. S1), the initialization of the South Atlantic SST and the accumulation of those signals in the southwest Indian Ocean appear to be important for the skillful prediction of the SST in the southwest Indian Ocean.

**Figure 2:**
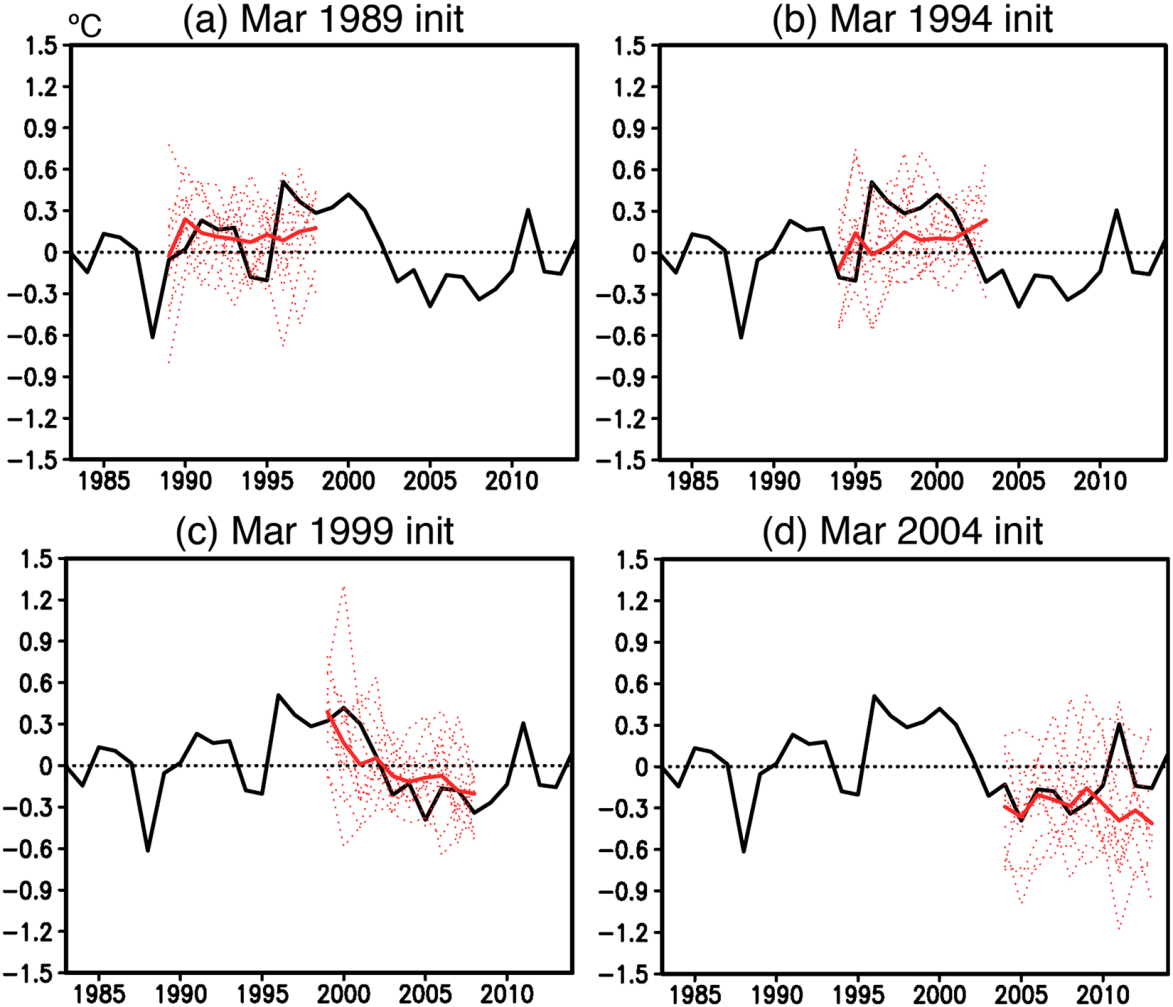
(**a**) Time series of yearly mean SWIO indices (in °C). Observation (black line) and reforecast experiments (red lines) initialized from March 1st 1989 are shown. The thick red line corresponds to 12 ensemble mean results, whereas the thin red dotted lines correspond to each ensemble member. (**b**–**d**) Same as in (**a**), but for the other reforecast experiments initialized from March 1st in 1994, 1999, and 2004, respectively.

In that context and to gain some useful insights into the high ACC in the southwest Indian Ocean, we have shown time series of individual reforecast results every 5 years since 1989 in addition to the observed and ensemble mean SWIO indices (Fig. 2). Considering a short period since 1982 when the decadal reforecast experiments were initiated, the reforecast results in the early 1980s are not considered here. The ensemble mean reforecast results initialized from March 1st 1989 show good agreement with the observation during 1989–1993, but they do not capture a sudden decrease in the observed SWIO index after 1994 (Fig. 2a). On the other hand, the experiments initialized from March 1st 1994 show persistence of the positive SWIO index, although the simulated amplitude in the ensemble mean SWIO index is weaker than the observed (Fig. 2b). Since some members capture the large decadal amplitude, the weaker ensemble mean may be partly due to under-performances of a few of the ensemble members. An increase in ensemble size is expected to improve the simulated amplitude.

The reforecast experiments initialized from March 1st 1999 (Fig. 2c) also have another noteworthy aspect. The ensemble mean SWIO index shows distinct change from a positive to negative phase. This agrees fairly well with the observed phase change, confirming a successful prediction of the cold phase in the southwest Indian Ocean. The negative SWIO index is also well captured in the reforecast results initiated from March 1st 2004 (Fig. 2d), but the model does not capture a rapid return to the normal state after 2010. These may be due to lack of initialization in the subsurface ocean and/or the atmosphere^21^, but the model using a simple SST-nudging initialization scheme shows good prediction skills in the warm period and its phase change to cold period in the southwest Indian Ocean.

### Predicted climate in warm and cold periods of the southwest Indian Ocean

**Figure 3:**
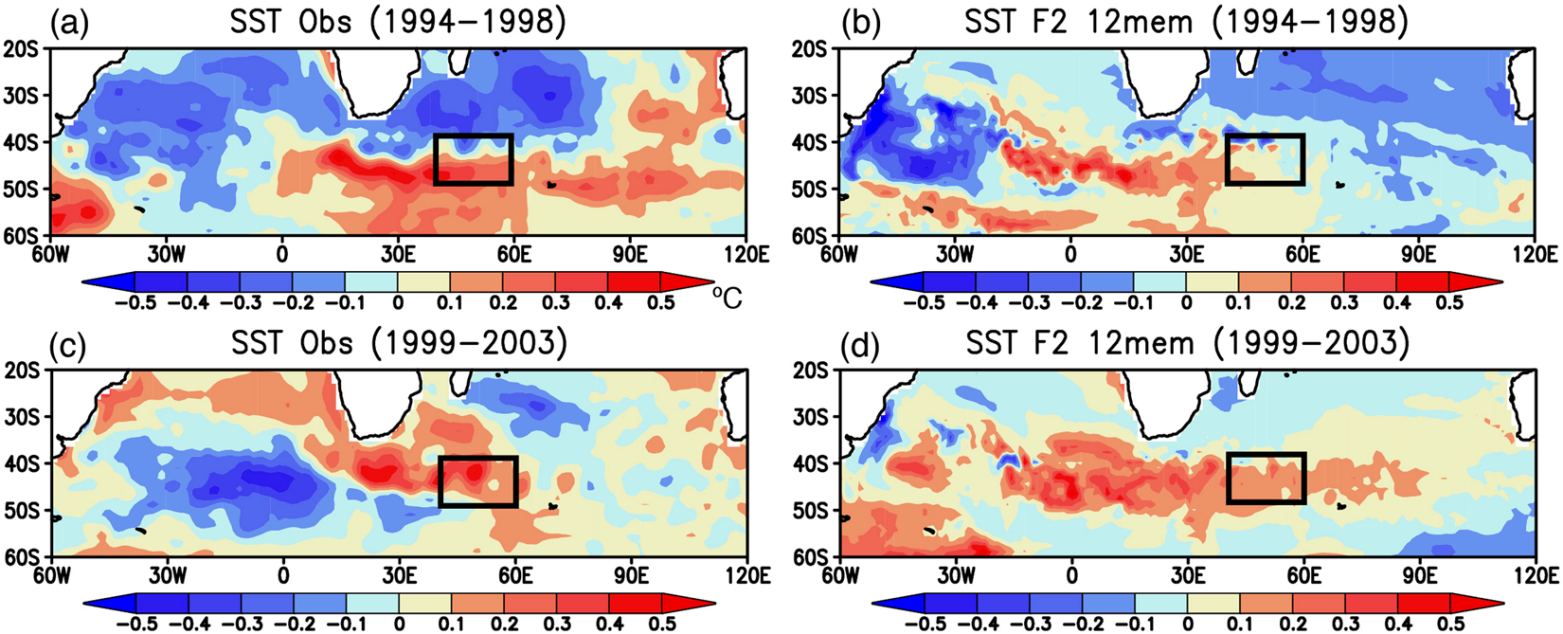
(**a**,**c**) 5-yr mean SST anomalies (in °C) observed during 1994–1998 and 1999–2003, respectively. The black boxes indicate the region where the SWIO index was calculated. (**b**,**d**) Same as in (**a**,**c**), but for the reforecast experiments initialized from March 1st 1994. Here we show 12 ensemble mean SST anomalies. The maps were generated using Grid Analysis and Display System (GrADS) Version 2.1.a3 (http://cola.gmu.edu/grads/downloads.php).

To further investigate physical mechanisms behind skillful prediction of the warm period and its phase change to the cold period in the southwest Indian Ocean, we calculated spatial patterns of SST anomalies during the warm period of 1994–2003 (Fig. 3). The observed anomalies during 1994–1998 show warm SST anomalies in the southwest Indian Ocean and cold SST anomalies south of Madagascar (Fig. 3a). This meridional dipole pattern of SST anomalies is well reproduced in the ensemble mean SST anomalies of reforecast experiments initialized from March 1st 1994 (Fig. 3b). The pattern correlation coefficient in the southern Indian Ocean (PCC_SIO) of interest between the observation and the model results is moderately high at 0.4. During 1999–2003, the observed dipole SST anomalies shift northward, accompanied by eastward propagation of cold SST anomalies in the South Atlantic (Fig. 3c). Although the reforecast results do not capture the cold SST anomalies in the South Atlantic, northward shift of dipole SST anomalies in the southern Indian Ocean are predicted (Fig. 3d; 0.28 PCC_SIO). This suggests that local air-sea interaction in the southwest Indian Ocean may amplify the decadal signal traveling from the South Atlantic to the southern Indian Ocean, as discussed in Morioka *et al*.^8^.

**Figure 4:**
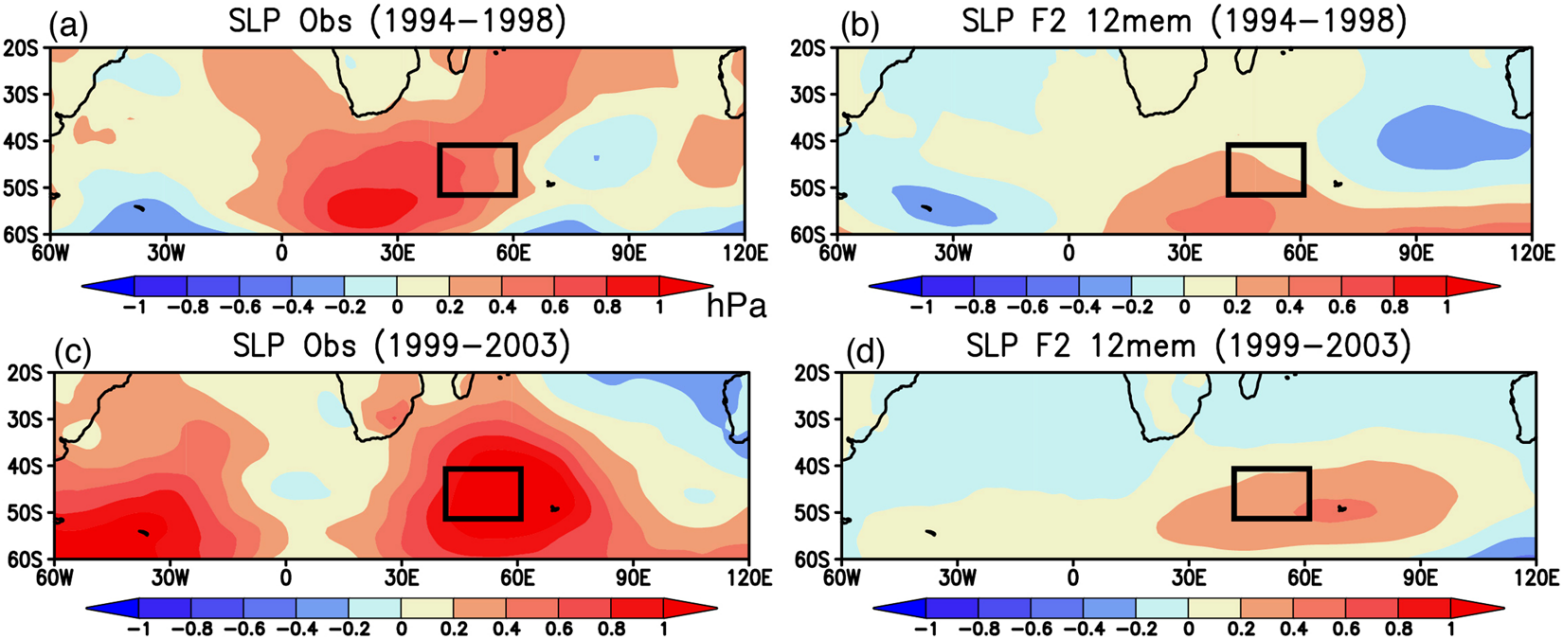
(**a**,**c**) 5-yr mean SLP anomalies (in hPa) observed during 1994–1998 and 1999–2003, respectively. The black boxes indicate the region where the SWIO index was calculated. (**b**,**d**) Same as in (**a**,**c**), but for the reforecast experiments initialized from March 1st 1994. Here we show 12 ensemble mean SLP anomalies. The maps were generated using Grid Analysis and Display System (GrADS) Version 2.1.a3 (http://cola.gmu.edu/grads/downloads.php).

A good agreement between the observation and the reforecast results is obtained for the SLP anomalies. During 1994–1998, the anticyclonic circulation anomalies appear south of southern Africa, accompanied by cyclonic circulation anomalies to the southwest (Fig. 4a). A series of positive and negative SLP anomalies is well reproduced in the reforecast experiments, except for a model bias of zonally elongated anomalies over the Indian sector of the Southern Ocean (Fig. 4b; 0.51 PCC_SIO). The observed anticyclonic circulation anomalies seem to move eastward to the southern Indian Ocean during 1999–2003, which is strongly accompanied by the positive SLP anomalies in the South Atlantic (Fig. 4c). Although the positive SLP anomalies in the South Atlantic are not well simulated, the reforecast results reasonably capture the positive SLP anomalies in the southern Indian Ocean (Fig. 4d; 0.73 PCC_SIO).

**Figure 5:**
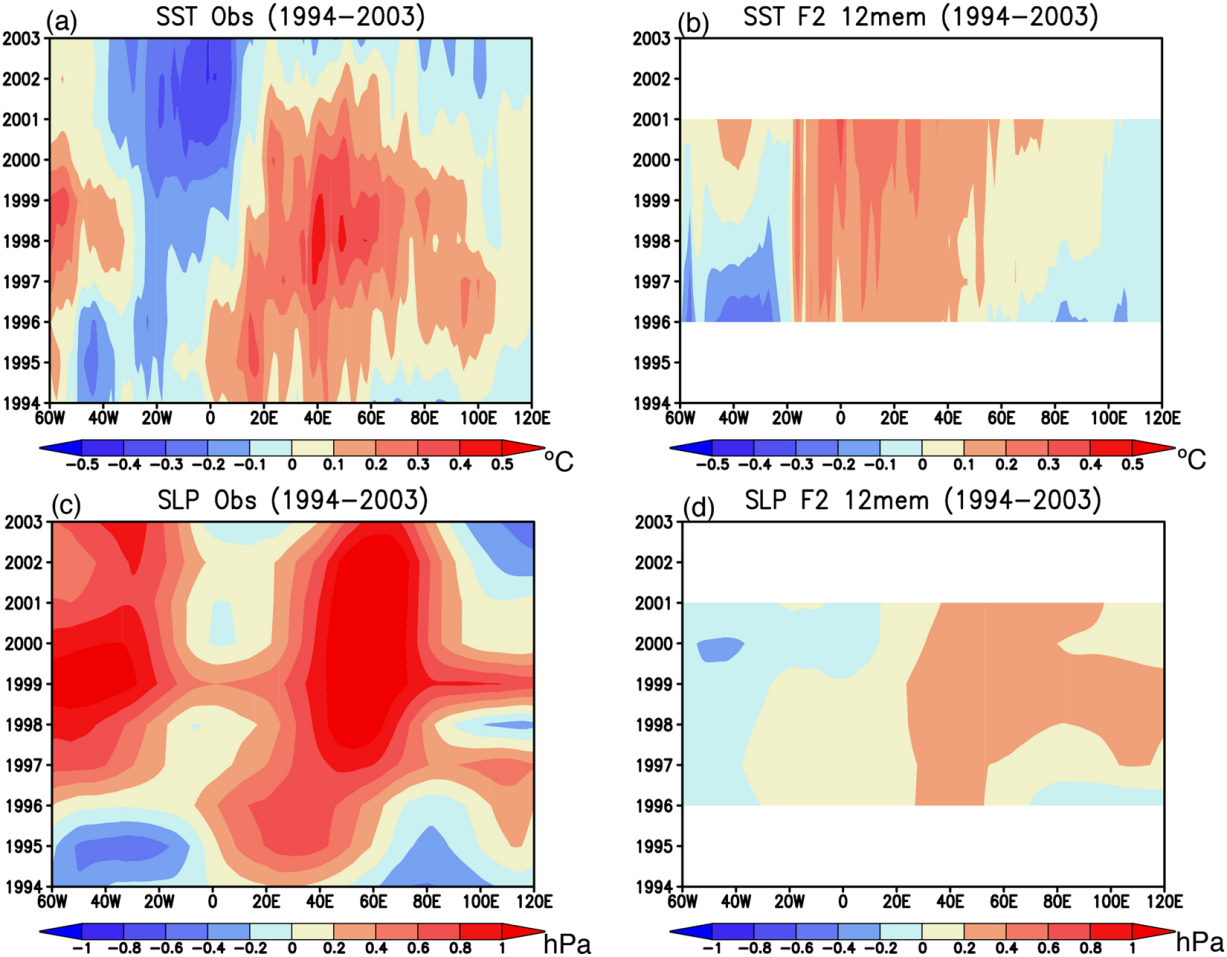
(**a**) Time-longitude section of 5-yr running mean SST anomalies (in °C) averaged in the longitudinal band of 40–50°S during 1994–2003. (**b**) Same as in (**a**), but for the reforecast experiments initialized from March 1st 1994. 12 ensemble mean results are used. (**c**,**d**) Same as in (**a**,**b**), but for the SLP anomalies (in hPa). The maps were generated using Grid Analysis and Display System (GrADS) Version 2.1.a3 (http://cola.gmu.edu/grads/downloads.php).

Since previous studies^8–10^ suggest both the warm SST and the high SLP anomalies in the southwest Indian Ocean originate from the South Atlantic and propagate eastward along the Antarctic Circumpolar Current, we describe time-longitude section of SST and SLP anomalies averaged in the latitudinal band of 40–50°S (Fig. 5). During 1994–2003, both the observed SST and SLP anomalies clearly show eastward propagation from the southeast Atlantic at 0°E to the southwest Indian Ocean at 60°E (Fig. 5a,c). The propagation speed is estimated to be approximately 2 cm s^−1^, consistent with the previous study^8^. The spatial pattern and eastward propagation of the warm SST and the high SLP anomalies are well captured in the reforecast results (Fig. 5b,d; 0.64 and 0.89 PCC_SIO), although the model does not reproduce the cold SST and high SLP anomalies in the southwest Atlantic during 1999–2003. Nevertheless, an analysis of the observation and the model results reveals that initialization of the SST anomalies in the southeast Atlantic is important for skillful prediction of the SST and SLP anomalies in the southwest Indian Ocean during the warm period of southern Indian Ocean.

**Figure 6:**
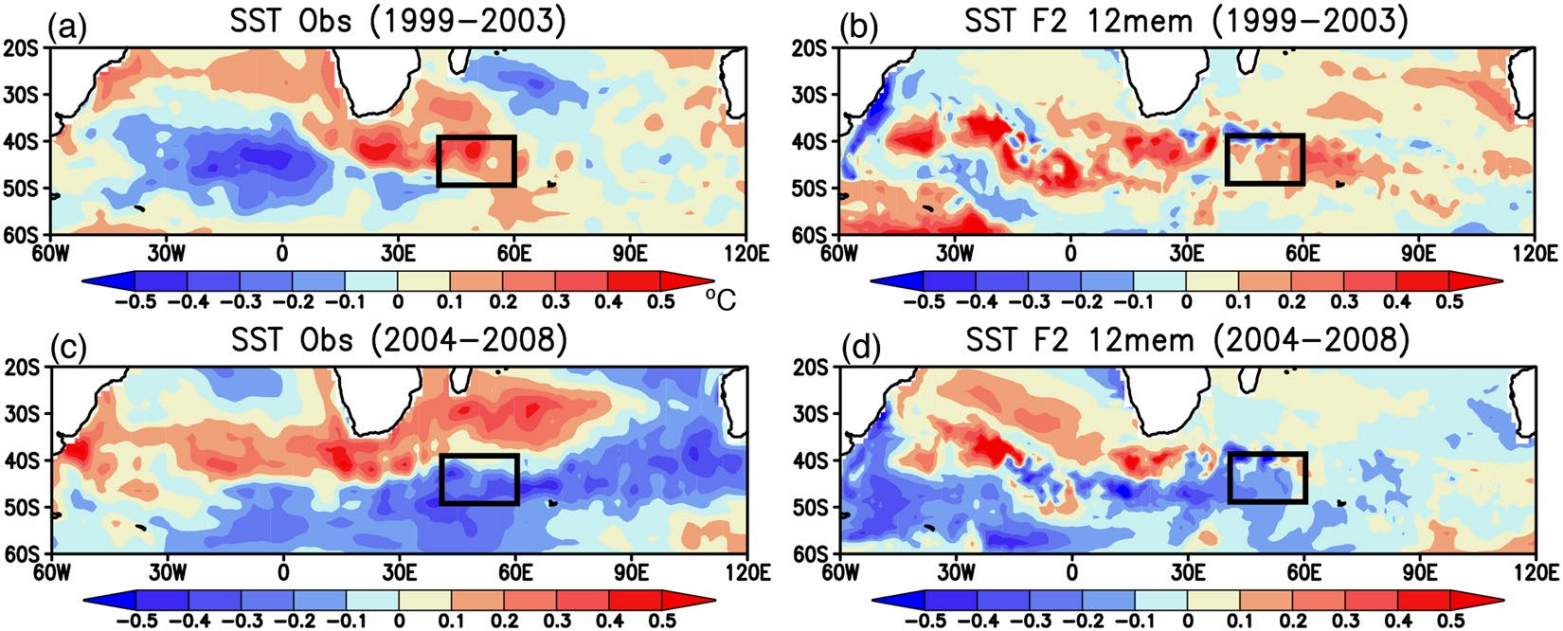
(**a**,**c**) 5-yr mean SST anomalies (in °C) observed during 1999–2003 and 2004–2008, respectively. The black boxes correspond to the region where the SWIO index was calculated. (**b**,**d**) Same as in (**a**,**c**), but for the reforecast experiments initialized from March 1st 1999. Here we show 12 ensemble mean SST anomalies. The maps were generated using Grid Analysis and Display System (GrADS) Version 2.1.a3 (http://cola.gmu.edu/grads/downloads.php).

**Figure 7:**
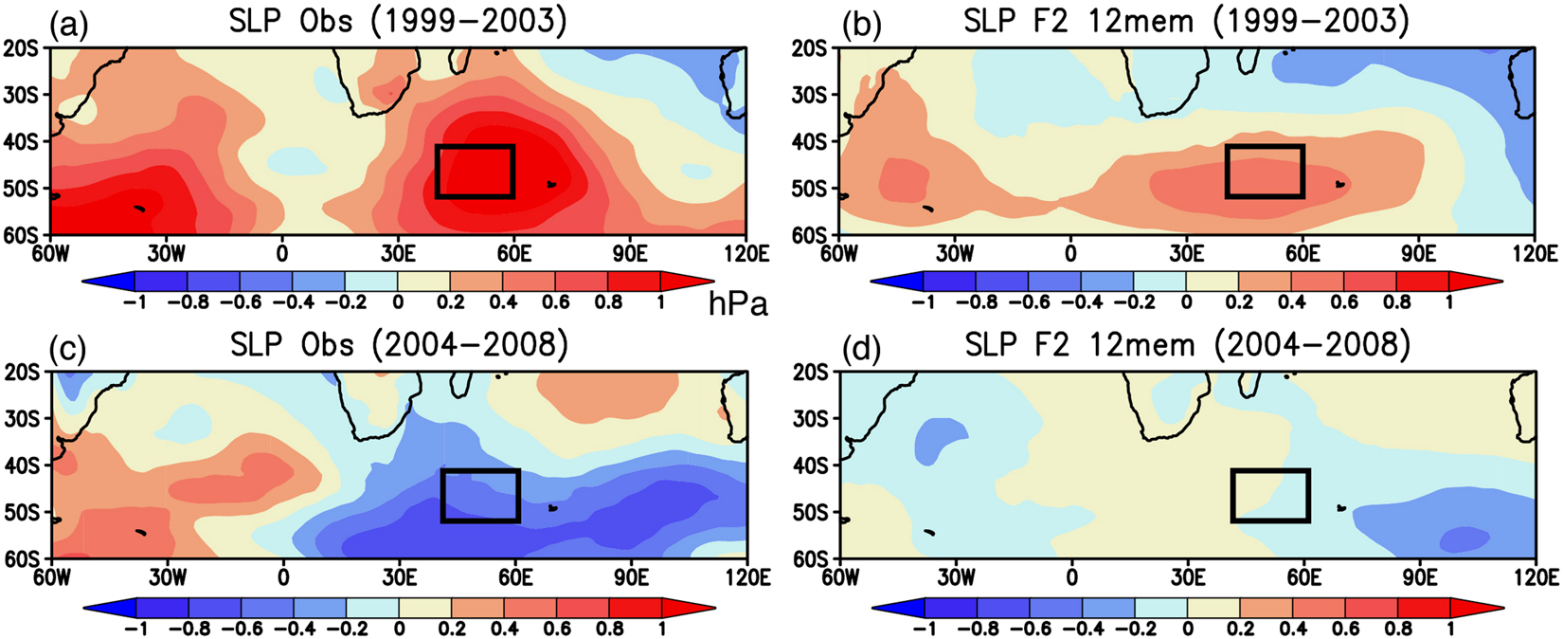
(**a**,**c**) 5-yr mean SLP anomalies (in hPa) observed during 1999–2003 and 2004–2008, respectively. The black boxes indicate the region where the SWIO index was calculated. (**b**,**d**) Same as in (**a**,**c**), but for the reforecast experiments initialized from March 1st 1999. Here we show 12 ensemble mean SLP anomalies. The maps were generated using Grid Analysis and Display System (GrADS) Version 2.1.a3 (http://cola.gmu.edu/grads/downloads.php).

On the other hand, roles of local air-sea interaction in the skillful prediction of southern Indian Ocean are found for the warm-cold phase change during 1999–2008. The observed cold SST anomalies in the South Atlantic during 1999–2003 appear to propagate eastward to the southern Indian Ocean during 2004–2008 (Fig. 6a,c). The reforecast results initialized from March 1st 1999 successfully reproduce cold SST anomalies in the southwest Indian Ocean during 2004–2008 (Fig. 6d; 0.46 PCC_SIO), although the model does not reproduce the cold SST anomalies observed in the South Atlantic (Fig. 6b; 0.28 PCC_SIO). This is complemented by a similar change in the SLP anomalies; the reforecast results well capture the observed phase change from the high to low SLP anomalies in the southwest Indian Ocean (Fig. 7b,d; 0.74 and 0.53 PCC_SIO), but the model does not capture the observed high SLP anomalies in the South Atlantic (Fig. 7c). These results suggest that local processes in addition to the remote influences from the South Atlantic may be responsible for the phase change to the cold period in the southwest Indian Ocean.

**Figure 8:**
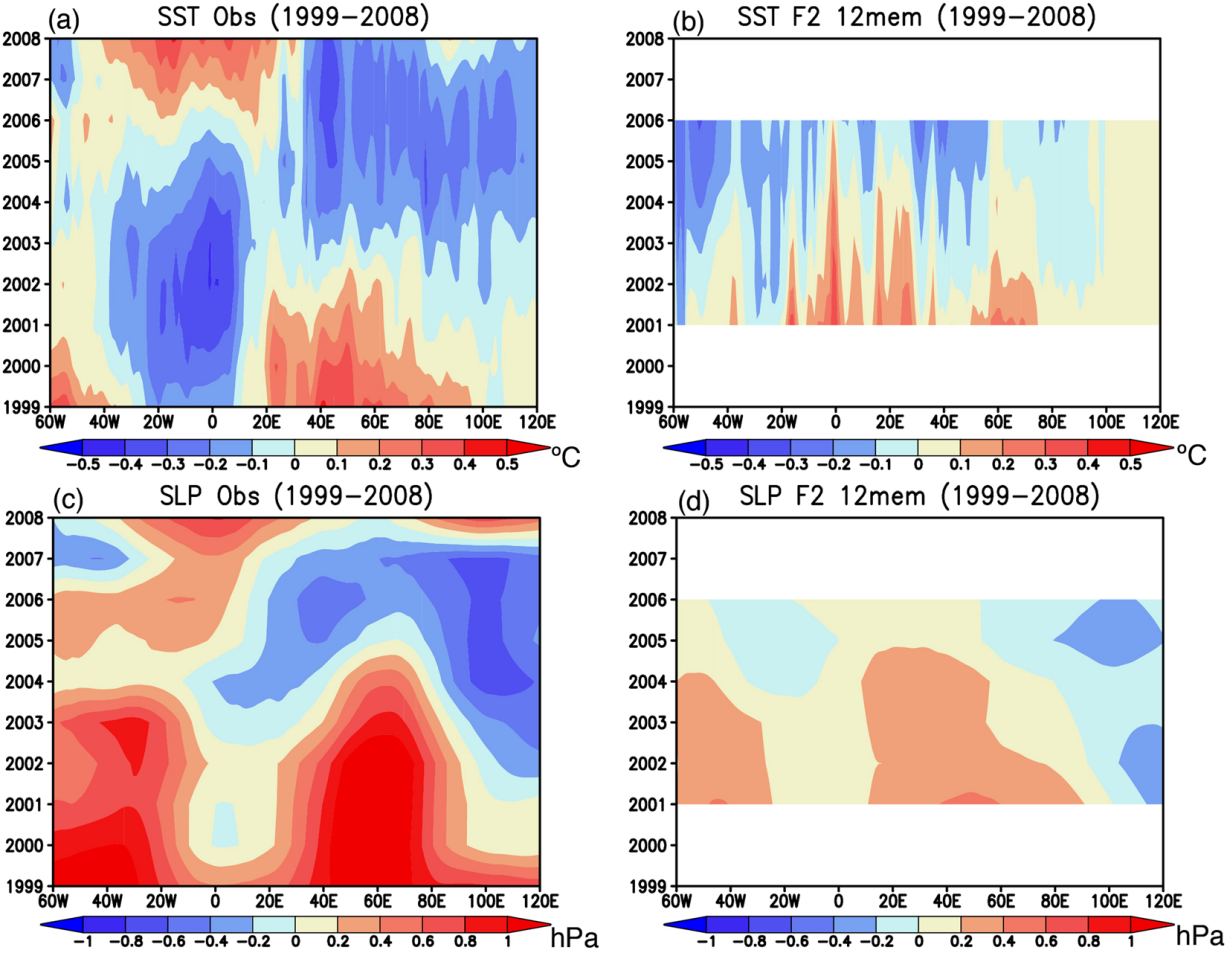
(**a**) Time-longitude section of 5-yr running mean SST anomalies (in °C) averaged in the longitudinal band of 40–50°S during 1999–2008. (**b**) Same as in (**a**), but for the reforecast experiments initialized from March 1st 1999. 12 ensemble mean results are used. (**c**,**d**) Same as in (**a**,**b**), but for the SLP anomalies (in hPa). The maps were generated using Grid Analysis and Display System (GrADS) Version 2.1.a3 (http://cola.gmu.edu/grads/downloads.php).

The time-longitude sections of the observed SST and SLP anomalies show weaker eastward propagation of anomalies from the South Atlantic at 0°E to the southwest Indian Ocean at 60°E (Fig. 8a,c) compared to those in the warm period (Fig. 5a,c). The reforecast results initialized from March 1st 1999 do not reproduce well the observed eastward propagation, rather the warm SST and high SLP anomalies in the southwest Indian Ocean appear to turn to the cold SST and low SLP anomalies independent from the signals in the South Atlantic (Fig. 8b,d; 0.40 and 0.71 PCC_SIO). This indicates that in the model, at least, the warm-cold phase change seems to be predicted through local processes in the southern Indian Ocean, rather than the remote influence from the South Atlantic. This result is consistent with previous studies claiming importance of local air-sea interaction in the decadal variability of the southern Indian Ocean^4,5^.

## Discussion

This study describes predictability of decadal climate variations in the southern Indian Ocean using a state-of-the-art CGCM. Initialized with a simple SST-nudging scheme, decadal reforecast experiments exhibit moderately high prediction skills of yearly mean surface temperature in the southwest Indian Ocean at 9 year lead time. The moderately high prediction skill, during the warm period (1999–2008) of the southern Indian Ocean, is attributed to successful prediction of eastward propagation of warm SST and high SLP anomalies from the South Atlantic, which was demonstrated in previous studies^8–10^. On the other hand, during the phase change from the warm to cold period (2004–2013), the local air-sea interaction in the southern Indian Ocean, as suggested in Reason *et al*.^4,5^, appears to be responsible for the skillful prediction of the phase change in the CGCM results.

The obtained results indicate both the remote and local processes are important for generating the decadal climate variability in the southern Indian Ocean, but the relative contributions of these processes remain to be investigated. Also, the present CGCM does not show predictability of decadal temperature variability in the South Atlantic^32,33^. Le Bars *et al*.^9^ suggested that in a high-resolution ocean general circulation model, internal ocean variability generated through eddy-mean interaction plays an important role in inducing decadal temperature variability in the South Atlantic. Since the CGCM used in this study has relatively lower resolution in the ocean, further improvement in initialization scheme such as adopting a subsurface ocean data assimilation scheme would be indispensable, but this is work in progress.

One may also wonder if the atmospheric teleconnection associated with the tropical Pacific decadal variability such as the Pacific-South American (PSA)^34^ pattern contributes to the phase change from warm to cold period in the southern Indian Ocean. However, global spatial patterns of the predicted SST and SLP anomalies do not show good agreement with those of the observation (see Supplementary Figs S2, S3). Rather, this may be partly responsible for lower prediction skills in the subtropical region (20–30°S) where the remote influences from the tropical decadal variability are more pronounced through changes in the atmospheric general circulations (i.e. Walker and Hadley Cells). In the southwest Indian Ocean (40–50°S), however, local air-sea interaction^4,5^ involving low-frequency variability in subsurface ocean plays an important role besides the remote processes. In particular, the strong SST fronts along the Agulhas Return Current provide an excellent background state that is known to exert local air-sea feedback^35,36^ strong enough to maintain the SST and SLP anomalies. On decadal timescale, the SST anomalies widely distributed along this region have potential in modulating the storm track activity through changes in the near-surface atmospheric stability (i.e. baroclinicity) and hence the large-scale atmospheric variability^36^.

This study provides compelling evidence that even the SST-nudging initialization scheme can be beneficial for skillful prediction of internally generated decadal climate variability in the southern Indian Ocean. The advantage of using the SST-nudging scheme is that one can extend decadal hindcast experiments over a longer period when the observed SST is available. However, for more realistic decadal climate prediction, external atmospheric forcing such as greenhouse gasses and aerosols should be taken into account, as discussed in much of the literature^11,12^. Since the present CGCM shows high prediction skills in the decadal climate variability in the southern Indian Ocean, further model experiments with and without extra-forcing would provide some useful insights into the relative importance of internal and external forcing in the decadal climate variability of the southern Indian Ocean.

The results described in this study have much implication for skillful prediction of decadal climate variation over southern Africa. The decadal climate variability in the southern Indian Ocean greatly influences the regional climate variability over southern Africa through modulation of the atmospheric circulation^1^. The high SLP anomalies accompanied with the warm SST anomalies in the southwest Indian Ocean, for example, during late 1990s and early 2000s provide more moisture toward southern Africa, leading to higher rainfall there. Skillful prediction of the decadal rainfall variability over the southern Africa requires good representation of the SST and SLP anomalies in the southwest Indian Ocean. Although there exists some disagreement in the atmospheric circulation anomalies between the observation and the model experiments, efforts on improving model physics and initialization scheme would lead to successful prediction of most if not all decadal climate variability over southern Africa. This would eventually benefit regional societies to mitigate potential risks such as consecutive droughts or floods by taking appropriate measures against the predicted climate in the near future.

## Methods

In this study, decadal reforecast experiments are tested using historical climate datasets. Atmospheric data are obtained from ERA-Interim atmospheric reanalysis^37^, whereas oceanic data are derived from Optimum Interpolation SST (OISST)^38^. Considering reliable ocean observation during the satellite period, we analyzed monthly mean variables with horizontal resolution of 1° × 1° during 1982–2015. For the above datasets, monthly anomalies were calculated with respect to monthly climatology.

The decadal reforecast experiments were carried out using the Scale Interaction Experiment-Frontier Research Center for Global Change 2 (SINTEX-F2)^39,40^ model, which is an upgraded version of SINTEX-F1^23,41^ model. This is a long-term extension of seasonal prediction system based on the SINTEX-F2 model^26^. The atmospheric component of the SINTEX-F2 is based on ECHAM5^42^, which was originally developed at ECMWF and has a parameterization package developed at the Max-Plank Institute for Meteorology. The ECHAM5 has 31 levels in the vertical on a T106 Gaussian grid. The oceanic component of the SINTEX-F2 is Nucleus for European Modeling of the Ocean (NEMO)^43^, which includes the Louvain-la-Neuve Sea Ice Model 2 (LIM2) sea ice model^44^ and has 0.5° × 0.5° horizontal resolution of ORCA configuration (ORCA05) with 31 levels in the vertical. The atmospheric and oceanic fields are exchanged every 2 hours with no flux correction by means of the Ocean Atmosphere Sea Ice Soil 3 (OASIS3) coupler^45^.

The SINTEX-F2 model was first spun up with monthly climatology of the observed SST during 1950–1981, then initialized with the realistic SST for each month of 1982–2016. Starting from every March 1 of 1982–2006, we performed decadal reforecast experiments with 12 different initial conditions via a simple SST-nudging scheme as follows: the model SST is strongly nudged to the observation by applying three large negative feedback values (−2400, −1200, and −800 W m^−2^ K^−1^) to the surface heat flux. Here we used two different SST datasets of the weekly OISSTv2^38^ with a horizontal resolution of 1° × 1° and the high-resolution daily NOAA OISST analysis^46^ with a horizontal resolution of 0.25° × 0.25°. Also, we adopted two different kinds of ocean vertical mixing scheme developed by Sasaki *et al*.^47^.

The ensemble mean of monthly climatology for the above 12 ensemble members was calculated over 10 years (i.e. 120 months) and the model drift (systematic error) is defined as difference in monthly climatologies between the model and the observation. A simple averaging technique was used in the calculation of ensemble mean. Monthly anomalies for each ensemble member were calculated by extracting the monthly observed climatology and the model drift from the total values.

## Electronic supplementary material

Supplementary information
