# Supplementary material for: Decadal climate predictability in the southern Indian Ocean captured by SINTEX-F using a simple SST-nudging scheme
Source: Sci Rep. 2018 Jan 26;8:1029. doi: 10.1038/s41598-018-19349-3 (PMC5786087; doi:10.1038/s41598-018-19349-3)
Supplement: Supplementary file 1 — Supplementary information [file 41598_2018_19349_MOESM1_ESM.pdf]

# Supplementary information for

# "Decadal climate predictability in the southern Indian Ocean captured by SINTEX-F using a simple SST-nudging scheme"

Yushi Morioka[1], Takeshi Doi[1], Swadhin K. Behera[1]

[1]*Application Laboratory, JAMSTEC, Yokohama, Japan*

Corresponding author: Dr. Yushi Morioka

E-mail: morioka@jamstec.go.jp

## Supplementary Figure Captions

**Figure S1:** The ACC of the 5-yr mean SST anomalies in the South Atlantic between the observation and reforecast experiments over 6-10 year lead times. Same as in Fig. 1b, the significant ACCs above the persistence values are colored. Here we used 12 ensemble mean SST anomalies for the model results. The map was generated using Grid Analysis and Display System (GrADS) Version 2.1.a3 (http://cola.gmu.edu/grads/downloads.php).

**Figure S2:** (a, c) 5-yr mean SST anomalies (in ºC) observed during 1999-2003 and 2004-2008, respectively. (b, d) Same as in (a, c), but for the reforecast experiments initialized from March 1st 1999. Here we show 12 ensemble mean SST anomalies. The maps were generated using Grid Analysis and Display System (GrADS) Version 2.1.a3 (http://cola.gmu.edu/grads/downloads.php).

**Figure S3:** (a, c) 5-yr mean SLP anomalies (in ºC) observed during 1999-2003 and 2004-2008, respectively. (b, d) Same as in (a, c), but for the reforecast experiments initialized from March 1st 1999. Here we show 12 ensemble mean SLP anomalies. The maps were generated using Grid Analysis and Display System (GrADS) Version 2.1.a3 (http://cola.gmu.edu/grads/downloads.php).

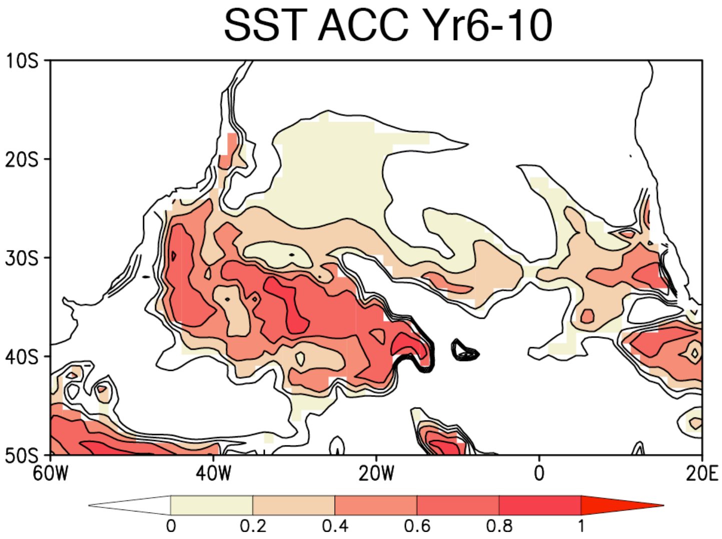


**Figure S1:** The ACC of the 5-yr mean SST anomalies in the South Atlantic between the observation and reforecast experiments over 6-10 year lead times. Same as in Fig. 1b, the significant ACCs above the persistence values are colored. Here we used 12 ensemble mean SST anomalies for the model results. The map was generated using Grid Analysis and Display System (GrADS) Version 2.1.a3 (http://cola.gmu.edu/grads/downloads.php).

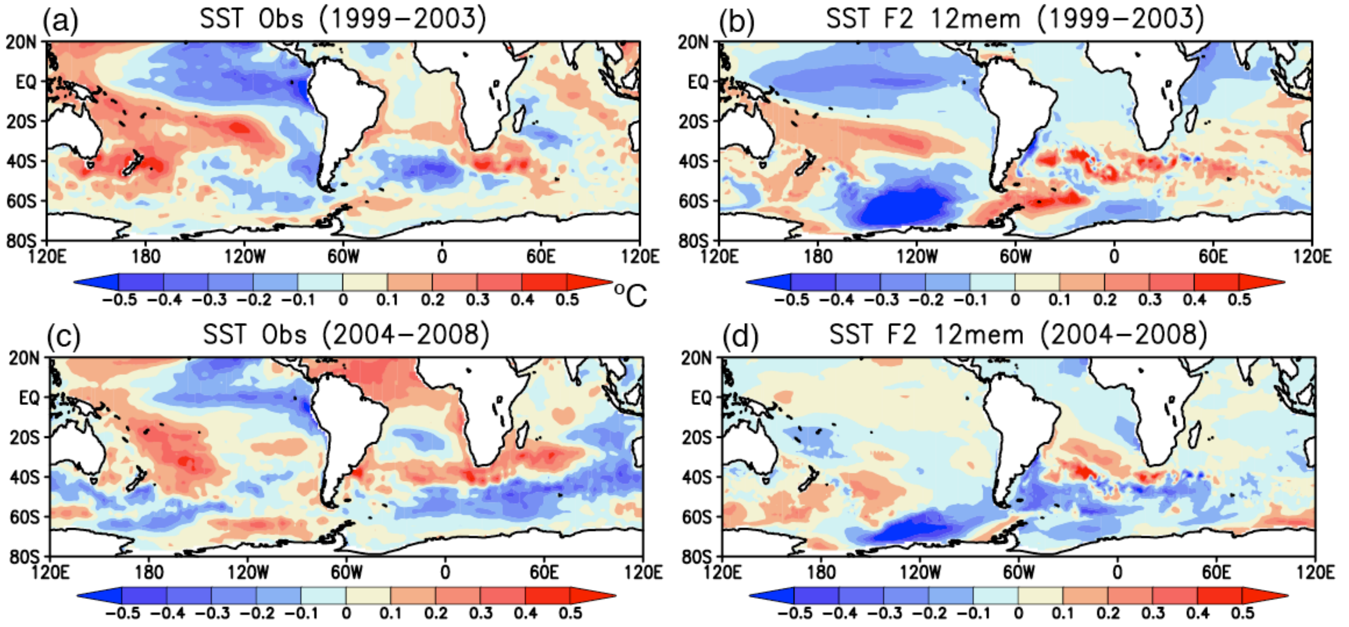


**Figure S2:** (a, c) 5-yr mean SST anomalies (in ºC) observed during 1999-2003 and 2004-2008, respectively. (b, d) Same as in (a, c), but for the reforecast experiments initialized from March 1st 1999. Here we show 12 ensemble mean SST anomalies. The maps were generated using Grid Analysis and Display System (GrADS) Version 2.1.a3 (http://cola.gmu.edu/grads/downloads.php).

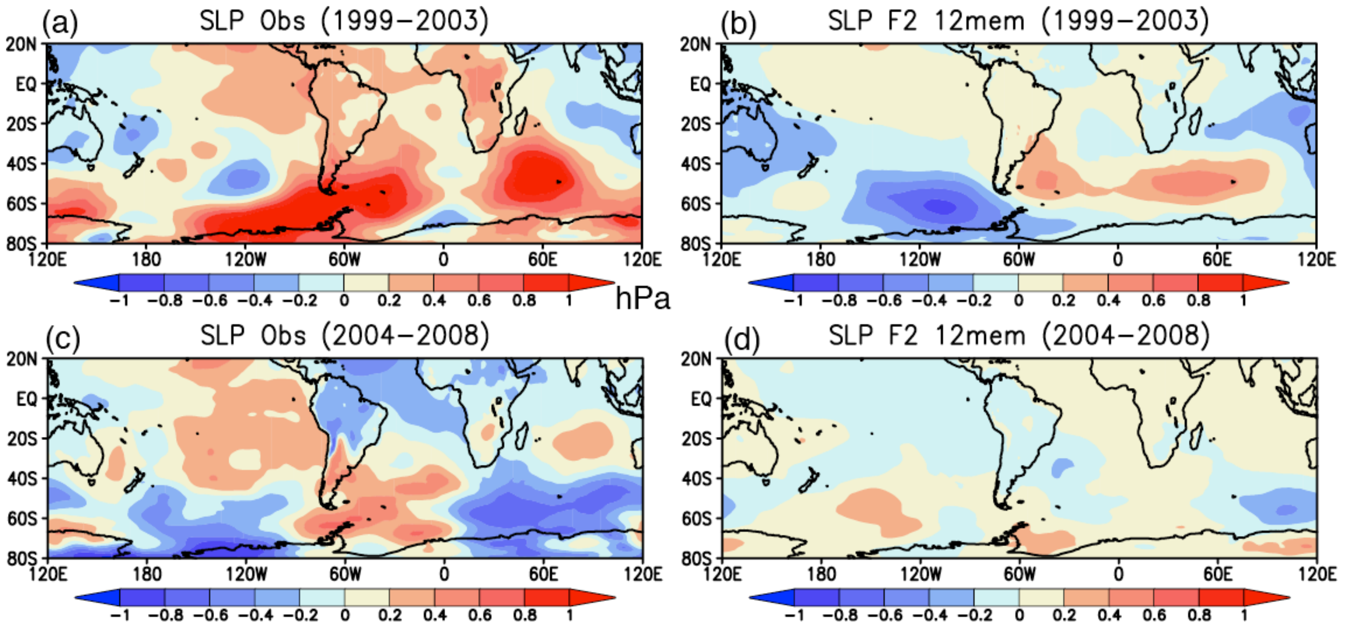


**Figure S3:** (a, c) 5-yr mean SLP anomalies (in ºC) observed during 1999-2003 and 2004-2008, respectively. (b, d) Same as in (a, c), but for the reforecast experiments initialized from March 1st 1999. Here we show 12 ensemble mean SLP anomalies. The maps were generated using Grid Analysis and Display System (GrADS) Version 2.1.a3 (http://cola.gmu.edu/grads/downloads.php).
